# Supplementary material for: An In Vitro Pilot Fermentation Study on the Impact of Chlorella pyrenoidosa on Gut Microbiome Composition and Metabolites in Healthy and Coeliac Subjects
Source: Molecules. 2021 Apr 16;26(8):2330. doi: 10.3390/molecules26082330 (PMC8072933; doi:10.3390/molecules26082330)
Supplement: Supplementary file 1 [file molecules-26-02330-s001.zip › molecules-1128557-supplementary.pdf]

**Table S1.** Nutritional composition of *Chlorella pyrenoidosa* (SunChlorella® ''A' Powder ).

| | | Current Supplement Facts | | % of NRV (Nutrient Reference Value* |
|---|---|---|---|---|
| | | /100g | /3g | |
| Moisture | g | 4.8 | 0.1 | |
| Protein | g | 54.7 | 1.6 | |
| Fat | g | 11.9 | 0.4 | |
| Ash | g | 7.2 | 0.2 | |
| Total carbohydrate | g | 21.4 | 0.6 | |
| Sugar | g | 8.9 | 0.3 | |
| Dietary fiber | g | 12.5 | 0.375 | |
| Energy | kcal | 387 | 12 | |
| **Elements** | | | | |
| Sodium | mg | 38.1 | 1.1 | |
| Phosphorus | g | 1.61 | 0.0483 | 7 |
| Iron | mg | 162.0 | 4.9 | 35 |
| Calcium | mg | 748.0 | 22.4 | |
| Potassium | mg | 991.0 | 29.7 | |
| Magnesium | mg | 357.0 | 10.7 | |
| Copper | mg | 0.20 | 0.01 | |
| Zinc | mg | 0.92 | 0.03 | |
| Iodine | mg | not detected | not detected | |
| Manganese | mg | 36 | 1.08 | 54 |
| **Nutrients** | | | | |
| Chlorophyll | mg | 2,100 | 63 | |
| Chlorophyll B | mg | not detected | not detected | |
| Vitamin A (retinol equivalents) | μg | 3,508 | 105 | 13 |
| Retinol | μg | not detected | not detected | |
| a-carotene | μg | 6,300 | 189 | |
| β-carotene | μg | 17,900 | 537 | |
| Vitamin B1 | mg | 1.54 | 0.05 | |
| Vitamin B2 | mg | 4.67 | 0.14 | 10 |
| Vitamin B6 | mg | 1.54 | 0.05 | |
| Vitamin B12 | mg | 0.23 | 0.07 | 276 |
| Vitamin C | mg | 12 | 0.36 | |
| Vitamin D | μg | 899 | 27 | 540 |
| Vitamin D (international unit) | IU | 36,000 | 1,080 | |
| Vitamin E | mg | 3.50 | 0.11 | |
| Vitamin K1 | μg | 865.0 | 26.0 | |
| Vitamin K2 | μg | not detected | not detected | |
| Folic acid | μg | 1.2 | 0.036 | 18 |
| Pantothenic acid | mg | 1.80 | 0.05 | |
| Biotin | μg | 210 | 6 | 13 |
| Inositol | mg | 228 | 7 | |
| Niacin equivalent | mg | 36.9 | 1.11 | 7 |
| Niacin (as nicotinic acid) | mg | 19.60 | 0.59 | |
| Tryptophan | g | 1.04 | 0.0312 | |
| Lutein | mg | 103 | 3 | |
| **Fatty acid composition** | | | | |
| 12:0 | | 0.2% | | |
| 14:0 | | 0.4% | | |
| 16:0 | | 15.0% | | |
| 16:1 | | 7.3% | | |
| 16:2 | | 3.9% | | |
| 16:3 | | 8.7% | | |

| | | Current Supplement Facts | | % of NRV (Nutrient Reference Value* |
|---|---|---|---|---|
| | | /100g | /3g | |
| 16:4 | | 2.5% | | |
| 17:0 | | | | |
| 18:0 | | 2.6% | | |
| 18:1 | | 9.6% | | |
| 18:2n-6 | | 20.1% | | |
| 18:3n-3 | | 19.6% | | |
| 20:0 | | 0.1% | | |
| 20:1 | | | | |
| 22:0 | | 0.2% | | |
| 24:0 | | 0.2% | | |
| Unknown | | 9.6% | | |
| n-3 Unsaturated fatty acids | g | 1.81 | 0.054 | |
| n-6 Unsaturated fatty acids | g | 1.85 | 0.056 | |
| **Amino acids** | | | | |
| Argentine | g | 3.16 | 0.0948 | |
| Lysine | g | 3.02 | 0.0906 | |
| Histidine | g | 0.99 | 0.0297 | |
| Phenylalanine | g | 2.54 | 0.0762 | |
| Tyrosine | g | 1.77 | 0.0531 | |
| Leucine | g | 4.30 | 0.1290 | |
| Isoleucine | g | 1.96 | 0.0588 | |
| Methionine | g | 1.22 | 0.0366 | |
| Valine | g | 3.04 | 0.0912 | |
| Alanine | g | 4.14 | 0.1242 | |
| Glycine | g | 2.94 | 0.0882 | |
| Proline | g | 2.48 | 0.0744 | |
| Glutamic acid | g | 5.87 | 0.1761 | |
| Serine | g | 2.12 | 0.0636 | |
| Threonine | g | 2.45 | 0.0735 | |
| Aspartic acid | g | 4.68 | 0.1404 | |
| Tryptophan | g | 1.04 | 0.0312 | |
| Cystine | g | 0.59 | 0.0177 | |

* Percentage of NRV (Nutritional Reference Value) defined per EU regulation 1169 / 2011. Calculated based on: Tsuboyama-Kasaoka N, Takimoto H, Ishimi Y. Comparison of Nutrient Reference Values for Food Labeling in Japan with CODEX Recommendations, Based on DRIs and Nutrient Intake in Japan. J Nutr Sci Vitaminol (Tokyo). 2019;65(1):102-105. doi: 10.3177/jnsv.65.102. PMID: 30814405.

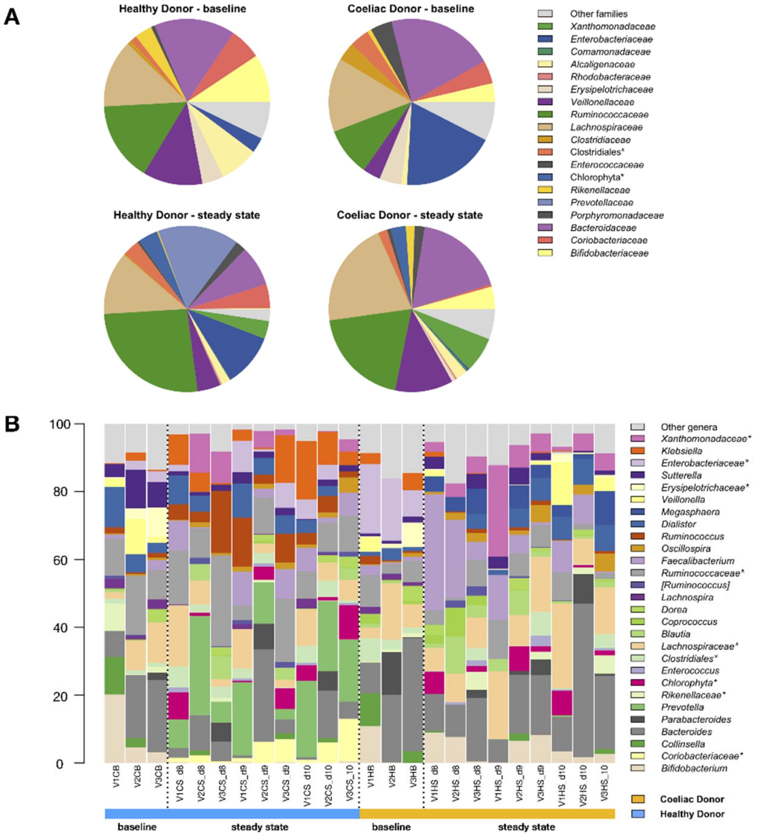


**Figure S1.** A schematic chart of the validated three-stage continuous.

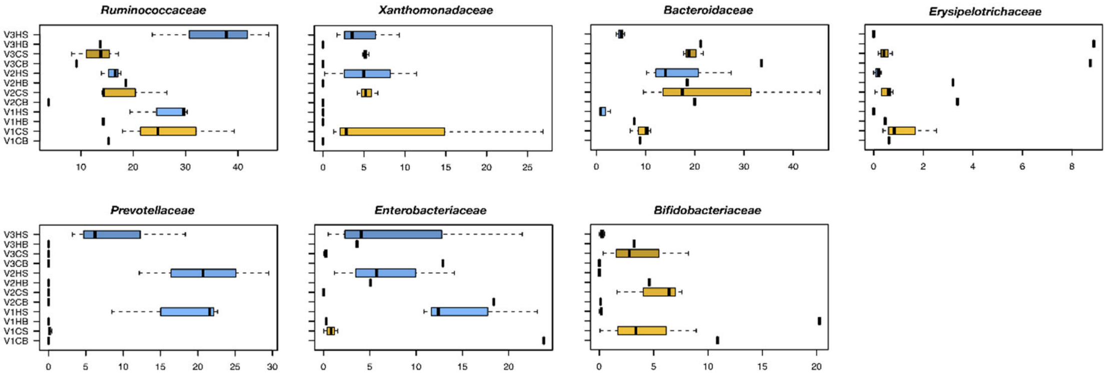


**Figure S2.** Profiles of faecal-derived microbial communities from a Coeliac (n = 1) and healthy donor (n = 1) in fermentation experiments in the presence of *C. pyrenoidosa* extract (Sun Chlorella® 'A' Powder). (A) Family-level relative abundance profiles at baseline and steady state (*i.e.* average values at day 8, 9, 10). (B) Genus-level relative abundance profiles are shown for each donor (Coeliac, C vs healthy, H), in the following order: at the baseline (B) and after *C. pyrenoidosa*addition(Sun Chlorella® 'A' Powder) upon reaching steady state (S, day 8, 9 and 10), in vessel (V) 1, 2 and 3 of the gut models. *, unclassified amplicon sequence variants reported at higher taxonomic level.

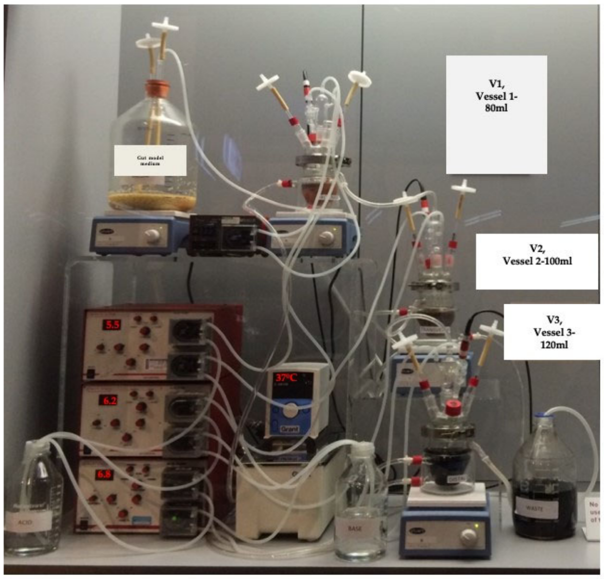


**Figure S3.** Family-level differences in the faecal-derived microbial communities between the Coeliac (n = 1) and healthy (n = 1) donor following fermentation with *C. pyrenoidosa* extract (Sun Chlorella® 'A' Powder). Boxplots showing the relative abundance distribution of bacterial families differentially represented among the study groups (vessel (V) 1, 2 and 3, inoculated with faeces from Coeliac (C) vs healthy (H) donors, at baseline (B) and steady state (S) after *C. pyrenoidosa*supplementation(Sun Chlorella® 'A' Powder) ($P \leq 0.1$, Kruskal-Wallis test).
